# Generation and Screening of a BAC Library from a Diploid Potato Clone to Unravel Durable Late Blight Resistance on Linkage Group IV

**DOI:** 10.1155/2007/51421

**Published:** 2007-12-03

**Authors:** Ingo Hein, Karen McLean, Boulos Chalhoub, Glenn J. Bryan

**Affiliations:** ^1^Genetics Programme, SCRI, Invergowrie, Dundee DD2 5DA, UK; ^2^UMR INRA-CNRS-UEVE de Recherches en Genomique Vegetale, 2 rue Gaston Cremieux, BP5708, 91057 Evry Cedex, France

## Abstract

We describe the construction and screening of a large insert genomic library from the diploid potato clone HB171(13) that has been shown to express durable quantitative field resistance to *Phytophthora infestans*, the causal agent of potato late blight disease. Integrated genetic mapping of the field resistance quantitative trait locus with markers developed from populations segregating for *Rpi-blb3, Rpi-abpt*, *R2*, and *R2*-like resistance, all located on linkage group IV, has positioned the field resistance QTL within the proximity of this *R* gene cluster. The library has been successfully screened with resistance gene analogues (RGA) potentially linked to the *R* gene cluster. Over 30 positive BAC clones were identified and confirmed by PCR and Southern hybridisations to harbour RGA-like sequences. In addition, BAC end sequencing of positive clones has corroborated two BAC clones with a very high level of nucleotide similarity to the RGA probes utilised.

## 1. INTRODUCTION

*Phytophthora infestans*, the causal agent of late blight disease in potato and responsible for the Irish potato famine in 1845-1846, remains, over 160 years later, the most serious disease of potatoes worldwide. A major quantitative trait locus (QTL) on potato linkage group (LG) IV, responsible for durable quantitative field resistance towards *P. infestans,* has been described for the tetraploid potato cultivar Stirling [1]. Stirling, which was released as a UK cultivar in 1991, has since been proven to express high levels of foliage and tuber resistance not only within the UK [2] but also in international field trials in Argentina, Canada, France, the Netherlands, USA, and Ecuador [3], the recently proposed origin of *P. infestans* [4]. A similar QTL for both foliage and tuber resistance has been described for a dihaploid potato clone PDH247, derived from the tetraploid breeding clone 8318(4), a close relative of Stirling [5]. The common parents of Stirling and 8318(4) were at least six backcrosses removed from the blight resistant Mexican wild hexaploid species *Solanum demissum,* the proposed origin of Stirling's field resistance [6].

An integrated genetic linkage map of potato LG IV has shown that major late blight resistance *R* genes such as *R2* from *S. demissum*, *R2*-like from an *S. demissum*-free pedigree, and *Rpi-abpt* and *Rpi-blb3* both from *S. bulbocastanum* also reside on this chromosome and form a single *R* gene cluster [7,8]. One AFLP marker, EATA/MACG_199, which was converted into the SCAR marker Th21, cosegregates closely with the above-mentioned *R* genes [7].

To physically clone the gene(s) contributing towards the field resistance QTL, a large insert genomic library in the form of a bacterial artificial chromosome (BAC) library was generated. The library originates from the SCRI diploid hybrid clone HB171(13), which scored 9.0 on a 1–9 scale of increasing resistance to a complex race of *P. infestans*. In terms of its origin, HB171(13) is an F1 clone derived from the cross between PDH247 (female) and DB226(70) (male). The second parent DB226(70) was the offspring of a pair cross between two diploid clones of *S. phureja* derived from a population which had been selected to tuberise in long days [9]. Importantly, HB171(13) was back-crossed with DB226(70) (male) in 1993 to produce the population HB193 which segregates for the field resistance QTL on LG IV [6]. In addition to the generation of a BAC library from HB171(13), we tested and mapped the marker Th21 on the diploid mapping population HB193 [HB171(13) × DB226(70)] to genetically position the field resistance QTL relative to the major *R* gene cluster described above.

## 2. MATERIAL AND METHODS

### 2.1. BAC library generation

For each extraction of high molecular weight DNA (HMW-DNA), 20 g of very young, only partially unfolded, potato leaves were harvested and flash frozen following dark-treatment for three days. To prepare HMW-DNA from potato suitable for the construction of BAC libraries, we have utilised a novel nuclei isolation procedure originally developed for woody perennial species such as raspberry [10]. The method is based on a modified buffer system including 4% (w/v) PVP-10 [11] and utilizes a combination of nylon filters and Percoll gradients to purify nuclei extracts prior to embedding in agarose plugs. All steps downstream of the HMW-DNA isolation, including restriction enzyme digestion, sizing, and cloning, were as described previously [10, 12]. Size fractionation of digested HMW-DNA was performed on a CHEF-Mapper apparatus (Bio-Rad) as described by Chalhoub et al. [12]. The commercially available BAC vector, plndigoBAC5-*Hin*dIII (Epicentre), was utilised for the cloning of DNA fragments. To estimate the insert size of BAC clones, BAC DNA was extracted from randomly selected colonies grown up for 24 hours at $37^{{}^{\circ}}$C in 1.5 ml 2xLB media containing chloramphenicol (12.5 μg/ml), using an alkaline lysis procedure [13]. Cloned genomic DNA was released by restriction enzyme digestion with *Not*I (New England Biolabs, Mass, USA) according to the manufacturer's recommendation. Digested products were separated on a 1% agarose gel (Gold Seakem) in 0.5x TBE utilising a CHEF-Mapper apparatus with the following parameters: pulse ramping 20 s constant, angle $120^{{}^{\circ}}$, current 6 V/cm, and run time 15 hours at $12^{{}^{\circ}}$C.

### 2.2. Probe generation

Primer sequences used to amplify a 481 bp portion of a nucleotide binding site (NBS) portion from a resistance gene analogue (RGA) (accession number CV286589) were 5′-TCATATGTGGATGACCAGGATAA-3′ and 5′-CTCTTTCCAGCCGACATCCT-3′. A second 500 bp RGA-NBS portion (accession number potato TC124441) was amplified by using the primers 5′-TGCAATTGTTGTATTGAGTGGA-3′ and 5′-GATACCTTTCTCCTTGACCATGA-3′. The estimated genome coverage was confirmed by hybridising the arrayed library with an LG IV specific SCAR marker, CT229 [7]. To assess the amount of chloroplast DNA contamination within the library, a 457 bp fragment of potato ribulosebisphosphate carboxylase/oxygenase (rbcL) (accession number M76402) was amplified utilising the primers 5′-CTGCAGGTACATGCGAAGAA-3′ and 5′-CCAAAGATCTCGGTCAGAGC-3′. DNA labelling and hybridisation were performed as described previously [14].

### 2.3. Mapping

Linkage map construction was performed using JoinMap 3.0 [15] as described previously [6]. Markers from different genetic maps were tested and mapped on the diploid population HB193 segregating for field resistance [6] and include STM3160 [1] and Th21 [7].

## 3. RESULTS

### 3.1. Generation of a BAC library suitable to positionally clone the gene(s) responsible for the large effect resistance QTL

**Figure 1:**
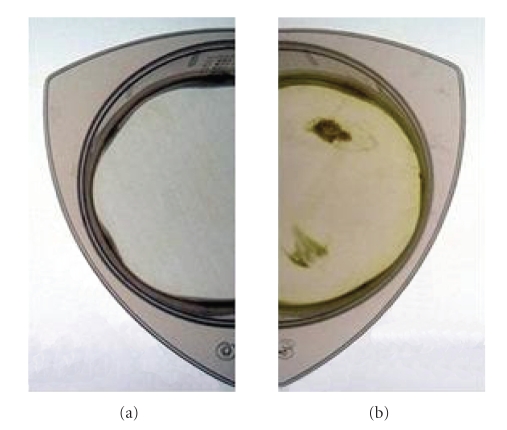
Filter (20 μm) used to purify nuclei suspension prior to embedding in agarose plugs, (a) before, and (b) after filtering.

The nuclei extraction method utilised for the generation of the potato BAC library had originally been developed for recalcitrant woody plant species such as raspberry and blackcurrant, which contain high levels of carbohydrates and polyphenolics [10]. One of the most crucial steps for raspberry nuclei extractions was the filtration of the homogenised plant tissue through 40 and 20 μm nylon meshes. Typically, a white precipitate formed on both the 40 and the 20 μm nylon meshes and turned brown within hours, suggesting that it contained carbohydrates and polyphenolics. Similarly, in potato, a mainly white precipitate formed on both meshes, which also turned brown, albeit to a lesser degree (Figure 1), suggesting that potato leaves also contain high levels of carbohydrates but fewer polyphenolics compared to raspberry.

**Figure 2:**
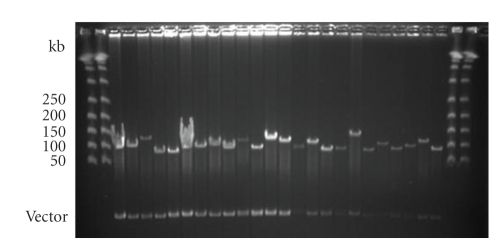
Sizing of 24 representative BAC clones. BAC clones were digested with *Not*I to release the cloned genomic insert, and sized on a 1% agarose gel (0.5x TBE) by separation on a CHEF gel.

The embedded nuclei contained high quality HMW-DNA suitable for restriction enzyme digestion (*Hin*dIII) and subsequent cloning. Currently, the library comprises approximately 280,000 individual clones. After analysing more than 100 BAC clones, the average insert size has been estimated to be about 100 kb (Figure 2), which totals nearly 28x genome equivalents. Approximately 4x coverage has been stored in 108 individual 384 well plates, which have been arrayed on three high density membranes comprising up to 18,432 clones per membrane (48×384 well plates). Multiple sets of filters have been generated for hybridisation screening. The remaining 24x genome coverage has been stored in 160 pools, each comprising approximately 1,500 recombinant BAC clones as described previously [16].

The estimated genome coverage on the arrayed filters has been assessed by hybridisation with a SCAR marker, CT229, located on LG IV [7]. The number of positively identified clones (over 20) significantly exceeded the estimated coverage (results not shown). The contamination of the library with chloroplast DNA was assessed by hybridising one filter with 18,432 individual clones to rbcL, the chloroplast coded large subunit of rubisco. Approximately 61 positive clones were identified, indicative of less than one percent chloroplast DNA contamination within the library (result not shown).

### 3.2. The large effect QTL for durable field resistance maps within the proximity of a major *R* gene cluster on LG IV

**Figure 3:**
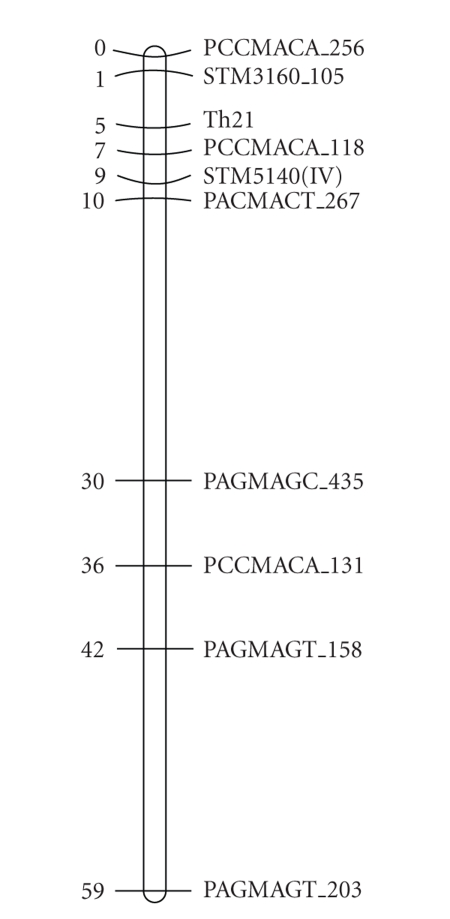
Genetic map of potato linkage group IV in the HB193 population including the markers STM3160 and Th21.

The large effect QTL for blight resistance mapped to LG IV in the HB193 population [HB171(13) × DB226(70)] and cosegregated within 10 cM of a microsatellite marker, STM5140 [6]. In this study, two additional markers, STM3160 [1] and Th21 [7], have been mapped to LG IV to improve the overall resolution of the region around the QTL and to position the QTL relative to *R2*, *R2*-like, *Rpi-abpt,* and *Rpi-blb3,* respectively. STM3160 maps to the top end (north) of chromosome 4 and Th21 maps between STM3160 and STM5140 (Figure 3).

### 3.3. Screening the BAC library with RGA derived probes from LG IV has identified numerous BAC clones comprising RGA-like sequences

**Figure 4:**
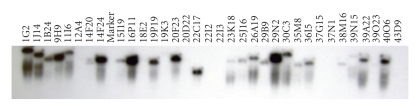
Southern hybridisation of 34 selected BAC clones with the probe 124441 derived from the NBS part of an RGA potentially linked to the field resistance QTL. The nomenclature describes the location of BAC clones in terms of storage plate and well position.

A previous study by Park et al. [7] had shown that a tomato BAC-end sequencing marker, TG370F, lies close (2.5 cM) to Th21. Interestingly, the corresponding tomato BAC clone (accession AF411807) harbours at least three RGAs. We designed two probes specific to the nucleotide binding side of the RGAs and utilised those to screen the arrayed library. Over 30 positive BAC clones were identified and confirmed by PCR and Southern hybridisations to harbour at least one or both NBS-RGA sequences. An example of a Southern confirming over twenty BAC clones from a selection of 34 is shown in Figure 4.

BAC-end sequencing of positive clones has identified two clones (1G2 and 30C3) with a nucleotide similarity greater than 75% to the NBS probes utilised. Furthermore, BlastX searches [17] of translated nucleotide sequences against the NCBI database has identified four additional clones with a high similarity to putative proteins located on the tomato BAC clone AF411807. These comprise 16P11 (e-value 3e-81), 19P19 (1e-13), 22C17 (2e-65), and 23K18 (1e-88).

## 4. DISCUSSION

Field resistance had previously been mapped in Stirling within 24 cM of STM5140 on an LG IV map of 105 cM in total length [1] and within 10 cM of STM5140 on an LG IV map of 61 cM in length for HB171(13) [6]. Another comparative analysis of this QTL for foliage resistance concluded that the QTL in Stirling was on the distal part of chromosome 4, in the same region as *R2* [18]. However, as STM5140 has not been mapped previously in a population segregating for *R2* or, conversely, markers closely linked to *R2* had not been mapped on a population segregating for field resistance, evidence for the regional proximity remained elusive. Our results (Figure 3) have shown that both STM5140 and STM3160 associated with field resistance, flank Th21, a SCAR marker cosegregating not only with *R2* and *R2*-like resistance but also with *Rpi*-*blb3* and *Rpi*-*abpt*. Despite the constraints of the current LG IV map in terms of limited population size (120 individual clones) and limited marker availability, the result supports the finding of Simko [18] and places field resistance potentially within close proximity of *R2* and other resistance genes. It is tantalising to speculate that this part of LG IV harbours a “super” *R* gene cluster with numerous resistance genes such as *R2*, *R2*-like, *Rpi-blb3*, and *Rpi-abpt,* and field resistance present as allelic variants in different potato accessions including *S. demissum*, *S. demissum*-free pedigrees, and *S. bulbocastanum*. Previous studies of *R* genes and RGAs distribution in many different plants species, including members of the family Solanaceae, have shown that they indeed form clusters [19–21]. In potato, genetic studies have shown that, for example, 14 out of 19 dominant *R* genes mediating resistance towards viruses, nematodes, and *P. infestans* are located in five hotspots throughout the potato genome [22]. One example comprises *Gpa*, *Grp1*, conferring resistance to potato cyst nematodes [23,24], *Nb* and *Rx2*, conferring resistance to Potato virus X [25,26], and *R1,* conferring resistance to *P. infestans* [27]; all located in one interval on chromosome 5. However, to substantiate this claim, further analysis is required and could, for example, comprise integrating STM5140 and STM3160 in populations segregating for *R2*, *R2*-like, *Rpi-blb3,* or *Rpi-abpt* resistance. In addition, comparative sequencing of this potential *R* gene super cluster combined with association genetics would shed light onto the complexity and selection pressure on the individual *R* genes. One tool required for this analysis is the BAC library generated in this study, which utilised HB171(13), the female parent from the HB193 population.

Previous to this study, BAC libraries did not exist for potato cultivars that exhibited durable quantitative field resistance to late blight. A primary goal was to generate such a resource to enable cloning of the gene(s) responsible for the resistance trait localised on LG IV. The generated library is of high quality and comprises approximately 4x the genome coverage on arrayed membranes and further 24x genome coverage stored in PCR-screenable pools of approximately 1500 recombinants per pool. Contamination of chloroplast DNA, as assessed by hybridisation with rbcL, is less than 1%, which highlights the efficiency of our method in eliminating contamination. Indeed, this is a significant improvement on a BAC library generated previously for the potato genotype RH, the male parent of a mapping population used to generate an ultradense genetic recombination map of potato [28]. BAC-end sequencing revealed up to 15% contamination with chloroplast DNA [29]. However, it was interesting to note that a hybridisation screening of the 4x arrayed library with the SCAR marker CT229 identified an excess of 20 positive clones, 5 times the expected amount. Potential explanation could be that CT229 is either not a single copy gene, as originally thought, or that the marker sequence has cross-hybridised unspecifically to other BAC clones. In addition, due to restriction enzyme bias of genetic regions, which are often manifested in different G/C contents, this part of the genome could indeed be overrepresented in the BAC library. Only a more detailed sequence-based analysis of clones that have hybridised to CT229 will be able to highlight the true reason for this result.

The BAC clones, positively identified in the hybridisation screen with the conserved NBS part of RGAs closely associated with a potential *R* gene super cluster on LG IV, present an invaluable tool to positionally clone the gene(s) responsible for the resistance QTL. BAC-end sequencing of over 30 positive clones has already identified six BAC clones (1G2, 30C3, 16P11, 19P19, 22C17, and 23K18) with either a high nucleotide- or amino acid-homology to the probes utilised, which is indicative of successful screening. Sequence information from these clones will aid the development of additional markers that are more tightly linked to the resistance QTL, and, furthermore, will feature in the construction of a physical BAC contig harbouring flanking QTL markers. As the sequencing of potato and its close relative tomato progresses [30], this BAC library, specifically developed to unravel the durable field resistance found in Stirling and the diploid potato clone HB171(13), will form an important tool for comparative genomics studies of a putative *R* gene super cluster on LG IV.
